# Sensor-Modality-Aware Human Activity Recognition with the Convolutional Tsetlin Machine: Interpretable and Resource-Efficient Neuro-Symbolic Learning

**DOI:** 10.3390/s26144482

**Published:** 2026-07-15

**Authors:** Olga Tarasyuk, Anatoliy Gorbenko, Oleksandr Gordieiev, Artem Akulynichev, Rishad Shafik, Alex Yakovlev

**Affiliations:** 1 School of Engineering, Newcastle University, Newcastle upon Tyne NE1 7RU, UK; rishad.shafik@newcastle.ac.uk (R.S.); alex.yakovlev@newcastle.ac.uk (A.Y.); 2 School of Built Environment, Engineering and Computing, Leeds Beckett University, Leeds LS1 3HE, UK; a.gorbenko@leedsbeckett.ac.uk (A.G.); o.gordieiev@leedsbeckett.ac.uk (O.G.); 3 Department of Infocommunication Engineering, Kharkiv National University of Radio Electronics, 61166 Kharkiv, Ukraine; artem.akulynichev@nure.ua

**Keywords:** human activity recognition, inertial signal modalities, neuro-symbolic machine learning, Tsetlin Machine, explainability, visualization, pattern recognition

## Abstract

Human activity recognition (HAR) based on smartphone and wearable sensor data is commonly addressed using statistical learning methods and deep neural networks that often provide strong predictive performance, but at the expense of limited interpretability and substantial computational and energy requirements. Such limitations reduce their suitability for deployment in practical sensing environments where model decisions must be transparent, verifiable and executable on resource-constrained devices. In this work, we investigate the Convolutional Tsetlin Machine (CTM) for multimodal HAR using only the raw inertial signals (9 × 128) of the UCI-HAR dataset, rather than its pre-computed 561-feature representation. The Tsetlin Machine is a novel neuro-symbolic machine learning approach that offers two important advantages over many conventional machine learning methods: (i) it learns logic-based decision rules that support human inspection and provide a transparent basis for analyzing model decisions, and (ii) it operates with comparatively low computational complexity, making it well suited to efficient and low-power on-device learning. The proposed study systematically analyses the contribution of different feature modalities by decomposing the inertial signals space into semantically defined subsets according to: (i) sensor source: accelerometer and gyroscope; (ii) signal group: gyroscope angular velocity, body and total acceleration (including gravity); (iii) coordinate axis: *x*, *y* and *z*. A separate CTM classifier was trained for each modality and its combinations in order to determine the relative discriminative value of each modality group for activity classification. In addition to predictive performance, the study emphasizes the interpretability of the CTM model ensured by expressing each decision in the form of propositional clauses, thereby enabling visualization and direct inspection of the modality-specific patterns supporting each activity class. Owing to its symbolic structure and modest computational demands, the CTM provides a principled framework for the design of explainable, resource-efficient and deployable HAR systems. The proposed work therefore contributes toward trustworthy multimodal sensing by jointly addressing predictive performance, interpretability and suitability for embedded and mobile platforms.

## 1. Introduction

Human activity recognition has become a central task in mobile health, sports analytics, assisted living and context-aware human–computer interaction. By using inertial sensors such as accelerometers, gyroscopes, GPS, etc., embedded in smartphones, smartwatches, fitness bands and other wearable devices, HAR systems can infer different types of activities (e.g., walking, sitting, standing, running, stair climbing, exercising, etc.) or abnormal motion events from continuous streams of body-motion data. Wearable sensor-based HAR offers important practical advantages as compared to other techniques. It is less intrusive, more privacy-preserving and less dependent on environmental lighting or viewpoint than, for instance, camera-based recognition methods, and is more suitable for continuous monitoring in everyday conditions. These characteristics make sensor-based HAR an important enabling technology for human-centered edge intelligence [1]: personalized healthcare, fitness analytics, remote rehabilitation, fall detection, etc. Recent surveys [2,3] further highlight explainable AI and TinyML/Edge-AI as two of the most active emerging directions in sensor-based HAR, with growing interest in models that combine on-device efficiency with human-interpretable decision making rather than treating accuracy as the sole evaluation criterion.

Machine learning (ML) has been fundamental to the development of HAR because the relationship between body-motion signals and semantic activities is highly nonlinear, user-dependent and sensitive to sensor placement, sampling rate, activity intensity and personal movement style. Earlier HAR systems often relied on handcrafted statistical and frequency-domain features extracted from raw inertial signals, followed by classical classifiers such as decision trees, support vector machines, random forests or shallow neural networks. More recent works have moved towards deep learning models that learn discriminative representations directly from raw or minimally processed signals [2,4]. Convolutional neural networks (CNNs), recurrent neural networks (RNNs), long short-term memory networks (LSTMs), attention mechanisms and transformer-based architectures have been investigated for wearable HAR. Deep learning has improved recognition accuracy across different benchmarks, although performance still remains affected by the used dataset, subject splits, sensor configuration and evaluation protocols.

Recent HAR studies increasingly combine local temporal feature extraction with sequence modeling and sensor fusion [5,6,7]. CNN-based models are commonly used to detect local signal patterns in multichannel inertial windows, while CNN-LSTM, CNN-GRU, attention-based and transformer models attempt to capture longer temporal dependencies. Multi-sensor fusion approaches have also been proposed to combine accelerometer, gyroscope, magnetometer and other wearable sensors and their signals. For example, the UC Fusion method [6] was introduced to merge unique and common features in wearable multi-sensor HAR, addressing the challenge of heterogeneous sensor fusion.

Recent HAR models report strong results on public datasets such as UCI-HAR, WISDM, PAMAP2, HAPT, OPPORTUNITY and related benchmarks, often achieving accuracy in the 90–97% range depending on the dataset, preprocessing technique and evaluation approach [2,7]. Despite this progress, there are limitations that restrict the deployment of high-performing HAR models in wearable and edge environments. First, many deep HAR models are trained offline and then deployed as fixed classifiers. This limits their ability to adapt to the characteristics of a specific user. In practice, the same activity may generate different accelerometer and gyroscope signatures depending on a user’s height, gait, age, strength, injury history, device position, fatigue level or exercise technique. A model trained on population-level data can therefore perform well under benchmark conditions but degrade when deployed continuously for an individual user. This is particularly problematic in healthcare, rehabilitation and sports applications, where the recognition model should ideally adapt to gradual changes in behavior, tiredness, recovery progress or detect abnormal movement patterns.

Second, on-device learning remains difficult for many deep learning models. While TinyML and Edge-AI techniques have advanced low-power inference, on-device learning imposes a stricter requirement: the device must not only perform the inference but also update the model incrementally from streaming sensor data. Continual HAR research identifies concept drift, new users, changing sensor positions and unlabeled streams among major barriers to long-term deployment [7,8,9]. Recent works, e.g., [10], further emphasize that streaming sensor distributions may change over time because of target-user or sensor-location variation, considerably impacting classification accuracy. These issues are especially challenging for low-cost wearable devices, where memory, energy and computational resources are limited.

Third, HAR systems often use multiple sensor modalities and signal channels without fully quantifying their individual contribution to classification performance. A typical inertial HAR setup may include total and/or body-acceleration and gyroscope signals across the *x*, *y*, and *z* axes. However, not all channels are equally informative for all activities. Some activities may be mainly characterized by translational acceleration, others by rotational motion or axis-specific temporal patterns. If redundant or weakly informative channels can be removed, sampled less frequently or processed with reduced complexity, the device may reduce sensing, memory, computation and energy costs.

This paper investigates the Convolutional Tsetlin Machine [11] as an interpretable and resource-efficient approach to HAR from raw accelerometer and gyroscope signals. The Tsetlin Machine [12] is a logic-based machine learning model that represents learnt patterns using propositional logic clauses. Instead of relying on dense floating-point matrix operations, it creates conjunctive clauses over Boolean input features, where each clause consists of selected literals and their negations, creating self-explainable, verifiable inference rules. The CTM extends this principle by applying clauses as convolution-like pattern detectors over local regions of structured input. Granmo et al. introduced the CTM as an interpretable alternative to CNNs, emphasizing that both learning and inference can be performed using Boolean inputs, clause-based pattern representations, and simple bit-level operations, while the learned convolutional filters remain directly interpretable as propositional logic expressions rather than opaque numerical kernels [11].

The CTM is particularly suitable for wearable HAR for three main reasons. First, its logic-based representation and non-gradient descent automata-based learning offer the potential for a low computational footprint and efficient inference, making it attractive for resource-constrained edge devices. Second, its learning mechanism may support inexpensive continuous on-device adaptation, allowing the model to be tailored to individual users over time. Such adaptation could improve classification accuracy and help the model respond to changes in movement style, exercise fatigue, rehabilitation progress or sensor placement. Third, the learned clauses/logic rules are inherently interpretable. They can be inspected as logical conditions over sensor-derived input features. This creates an opportunity to reveal distinctive activity patterns and to identify which sensors, axes, and temporal regions contribute to recognition decisions.

The motivation of this work is therefore to move beyond accuracy-only HAR evaluation and study a model that is simultaneously accurate, interpretable, modality-aware and well-suited to resource-constrained deployment and on-device learning based on its memory footprint and computational characteristics. The paper evaluates CTM classification performance under different sensor and signal configurations, examines the trade-off between recognition accuracy and resource usage and investigates the interpretability of learned clauses as activity-specific signal patterns.

Through this, the work contributes to the design of HAR systems that are better aligned with the requirements of personalized, interpretable and resource-efficient on-device intelligence.

## 2. Materials and Methods

### 2.1. Human Activity Recognition Datasets

Several public datasets, including UCI-HAR, PAMAP2, WISDM, OPPORTUNITY and HAPT, have become standard benchmarks for sensor-based human activity recognition [2,13,14,15,16]. They differ in sensor configuration and placement, sampling rate, number of subjects, activity set and degree of experimental control. These inconsistencies preclude a direct, like-for-like comparison of the performance of different ML models across datasets without additional processing to align the sensor data and activities. For this reason, the UCI Human Activity Recognition Using Smartphones (UCI-HAR) dataset [13] was selected as the initial benchmark for this study. It remains one of the most widely used, well-established and technically suitable benchmarks, which facilitates qualitative comparison with the broad existing literature. In addition to 561 postprocessed features, the dataset provides raw, time-synchronized accelerometer and gyroscope windows of a fixed 128-sample length that directly match the patch-based input representation of the Convolutional Tsetlin Machine and are the primary data used in this work, requiring no additional resampling or segmentation. Larger and more sensor- and activity-rich multimodal datasets such as WISDM, PAMAP2 and OPPORTUNITY are less standardized and contain missing data. These were judged better suited to a follow-up study for testing generality across body placements and sensor configurations once the CTM-based methodology (patch-size selection, Booleanization strategy, explainability and modality decomposition) had first been established and validated on a single, well-controlled benchmark.

UCI-HAR dataset contains recordings from 30 volunteers aged 19–48 performing six activities: WALKING, WALKING_UPSTAIRS, WALKING_DOWNSTAIRS, SITTING, STANDING and LAYING, while wearing a Samsung Galaxy S II smartphone on the waist [13]. Its embedded accelerometer and gyroscope captured 3-axis linear acceleration and 3-axis angular velocity at 50 Hz. From the total acceleration signal, the body acceleration was also obtained by separating out the gravitational component using a Butterworth low-pass filter with a 0.3 Hz cutoff. The raw signals were segmented into fixed-width windows of 2.56 s, corresponding to 128 readings per window with 50% overlap, which naturally matches the patch-based processing approach of the Convolutional Tsetlin Machine. The dataset includes both dynamic locomotion classes and static postures, enabling evaluation of whether CTM clause patterns can distinguish between strongly dynamic activities, such as walking and stair movement, and more subtle posture-related classes, such as sitting, standing and laying. In the experiments, we used the original UCI-HAR subject-independent train/test split with 7352 training samples and 2947 test samples, ensuring that subjects in the test set were not used during model training.

### 2.2. Problem Formulation

We first formalize the problem addressed in this work. Let $S_{i}\left(t\right)$ for i=1,…,N and t=1,…,W denote the value of the *i*-th inertial sensor channel at discrete time step *t* within an input window where N=9 (*body_acc_x/y/z*, *total_acc_x/y/z*, *body_gyro_x/y/z*) and W=128 samples per window for the UCI-HAR dataset.

The HAR task is therefore to learn mapping:

$$f:{\mathbb{R}}^{\left(N\times W\right)}\to \left\{0,\ldots ,M-1\right\}$$

from a window $S=\left[S_{i}\left(t\right)\right]$ to one of the M activity labels Y, where M=6 for UCI-HAR and Y∈ {*WALKING*, *WALKING_UPSTAIRS*, *WALKING_DOWNSTAIRS*, *SITTING*, *STANDING*, *LAYING*}.

Because the Convolutional Tsetlin Machine used in this study (see Section 2.3) operates on Boolean inputs, i.e., literals that are either *True* (1) or *False* (0), *S* is first needs to be mapped by a Booleanization function *g* to a binary array $X=g\left(S\right)\in {\left\{0,1\right\}}^{\left(N\times W\cdot B\right)}$, where B is the number of thresholds per channel (Section 2.4); the corresponding input literal vector, which also includes the associated negated literals ¬X is therefore $L=\left\{X,\neg X\right\}\in {\left\{0,1\right\}}^{\left(2N\times W\cdot B\right)}$. The CTM then learns, for each class m=0,…,M−1 and clause index j=0,…,C−1, a clause function $c_{j}^{m}\left(L\right)\in \left\{0,1\right\}$ expressed as a conjunction of a learned subset of the literals in *L* (restricted to a patch of size N×P·B,P≤W, for the convolutional variant), and aggregates these into a class sum $v_{m}\left(L\right)=\sum_{j}^{C}p_{j}c_{j}^{m}\left(L\right)$, where $p_{j}\in \left\{+1,-1\right\}$ is the polarity of clause *j*. The predicted label is $\hat{Y}={\mathrm{argmax}}_{m}v_{m}\left(L\right)$.

The rest of the paper is organized as follows. Section 2.3 and Section 2.4 describe the automata-driven learning approach and clause-based inference employed by the Tsetlin Machine, together with the way the data Booleanization *g* is realized, while Section 2.5 discusses the CTM’s hyperparameters and justifies their choice. Section 3 reports the model’s predictive performance *f* and resource footprint under different choices of channel subsets/modalities *N*, size of the patch *P* and Booleanization granularity *B*; it also visualizes and interprets the CTM’s clause-based inference and analyses the importance and impact of the different sensor modalities on classification performance. Section 4 discusses the major findings and Section 5 concludes the paper.

### 2.3. Machine Learning with Interpretable Tsetlin Machine

**Figure 1:**
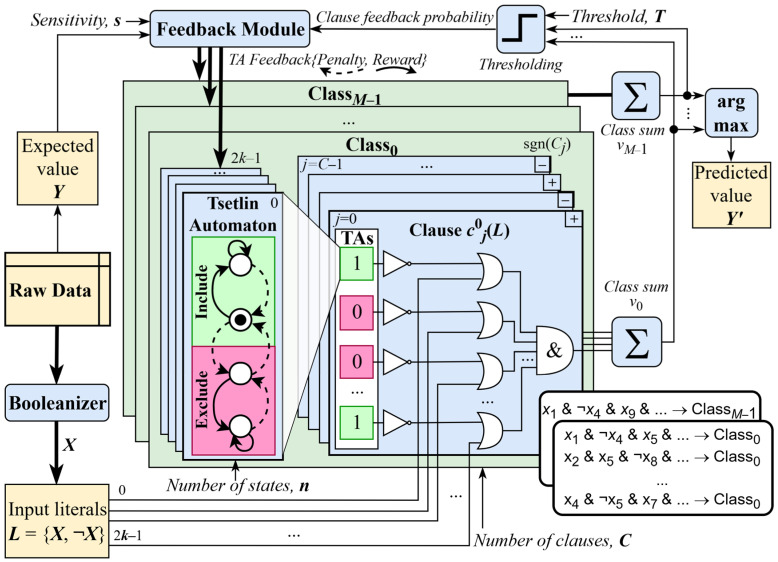
Basic TM architecture with cooperative automata and logic-based inference. TM’s inference is based on an ensemble of propositional clauses *C*, which collectively (through the voting of per-class clause sums) classify input data samples. Each clause is a conjunction of selected Boolean input literals *L* whose inclusion or exclusion is governed by dedicated Tsetlin Automata.

**Figure 2:**
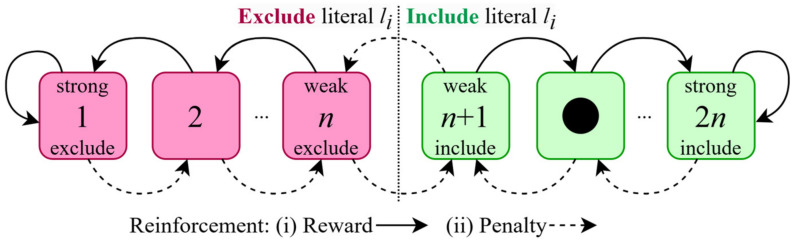
States and transitions of the Tsetlin Automaton, a two-action finite-state automaton, which governs the inclusion or exclusion of the linked Boolean literal into a related conjunctive clause. The TM starts training with all Tsetlin Automata initialized in the weak exclude state.

Tsetlin Machine is a type of machine learning algorithm that leverages the collective behavior of learning automata and bases its inference on interpretable logic-based rules, specifically conjunctive clauses [12,17]. These clauses logically link together selected input features, thereby creating distinct patterns that represent different classes, enabling TM to make transparent and interpretable predictions of human activity. The underpinning of the Tsetlin Machine is the Tsetlin Automata (TA), which originates from the research on collective behavior of learning automata by Tsetlin [18] and Varshavsky [19] and further developed by Narendra in [20]. Granmo incorporated propositional logic and a game-theoretic bandit-driven approach to orchestrate the collective behavior of TAs and formulate logic statements used for inference through group voting [12]. Thus, a collection of TAs, each making one decision, combined through NOT and AND operations to form logic propositions—clauses that are used to solve classification tasks—is referred to as the Tsetlin Machine, whose architecture is illustrated in Figure 1. TM’s input pipeline (Figure 1) begins by converting raw data into binary features through a Booleanizer, which is a preprocessing stage rather than part of the TM itself. Each resulting feature takes a Boolean value of either *True* (1) or *False* (0). These features and their negations form the input literals $L=\left\{X,\neg X\right\}$, which constitute the common input to every conjunctive clause in the machine. The core computational units are Tsetlin Automata (Figure 2). Each TA is a two-action finite-state automaton that decides whether the literal it controls is *included* in or *excluded* from a clause: the lower half of its 2*n* states corresponds to the *Exclude* action and the upper half to the *Include* action, with reward and penalty signals moving the automaton deeper into, or out of, each region. A group of TAs (one per literal) collectively defines a single conjunctive clause $c_{j}^{m}\left(L\right)$, which evaluates to *True* (1) only when all its included literals are satisfied by the input; equivalently, it implements a logical AND over the selected literals. In this way, each clause learns to recognize a specific sub-pattern in the data, and, as shown by the example rules in Figure 1 (e.g., $x_{1}\&\neg x_{4}\&x_{5}\&\;\ldots \to Class_{0}$); these clauses are directly readable as logical expressions, which is the source of the TM’s interpretability.

Clauses are organized per class: each of the M classes $\left(Class_{0},\ldots ,Class_{M-1}\right)$ is assigned its own bank of clauses $c_{j}^{m},j=0\ldots C-1,m=0\ldots M-1$. Within a class, clauses are given alternating polarities ($p_{j}^{m}=sgn\left(c_{j}^{m}\right)$, shown as ‘+’ and ‘−’ in the figure): positive clauses vote *for* the class, and negative clauses vote *against* it. The votes of all clauses belonging to a class are accumulated by a summation unit Σ to produce a clause voting sum $v_{m}$, i.e., a class score. During inference, an input sample is evaluated against all clauses of all classes. Each satisfied clause contributes a vote to its corresponding class, while unsatisfied clauses abstain. The final prediction $\hat{Y}$ is obtained by taking the argmax over the per-class sums $v_{m}$, thus selecting the class receiving the highest aggregated clause vote.

Learning is handled by the Feedback Module, which operates only during training. It compares the predicted clause output against the expected value Y and issues reward or penalty signals to the TAs according to rules described in [12], driving them toward *Include* or *Exclude* decisions (Figure 2).

This feedback is stochastic and threshold-regulated: the probability of reinforcing a given clause *j* depends on the voting threshold *T* and the learning sensitivity *s* (two major TM hyperparameters in addition to the number of clauses *C* and the number of TA’s states 2*n*), and it decreases as the clause voting sum $v_{m}$ approaches the target *T*. Through repeated feedback over the training data, the TAs converge on a configuration of clauses that jointly discriminate between the classes, without gradient descent or floating-point weight updates.

The CTM [11] extends the base TM by applying its conjunctive clauses locally rather than to the entire input at once. Each clause acts as a convolution-like pattern detector: it scans each input sample *S* (of dimension 9 sensor channels × 128 samples for UCI-HAR) using a sliding patch $p\in {\left\{0,1\right\}}^{2N\times P\cdot B}$, where P is the patch length, and *N* plays the role of a patch width to slide the patch simultaneously across all sensor channels. For instance, for the one-threshold representation (i.e., B=1) and P=114, the patch contains 9 × 2 × 114 Boolean features (including negated values). During inference, each clause is evaluated over all valid temporal patch positions (for the above example, the number of such positions (W−P) is equal to 14) and outputs *True* (1), contributing to the vote score of its class, if its selected literals are satisfied in at least one patch position.

A formal, detailed description of the TM and CTM, as well as clause-based inference method and TA feedback rules, is given in [11,12], while [17] compares the TM with DNNs and discusses the core hyperparameters *C*, *T*, *s* and 2*n* and their optimization.

TM is an actively evolving field of neuro-symbolic research where novel architectures and training methods are being developed and becoming available via the GitHub repository [21]. Here, the Python (v. 3.14) implementation of the *MultiClassConvolutionalTsetlinMachine2D* from the *pyTsetlinMachineParallel* package (v. 0.2.1) was used. The code is available at https://github.com/cair/pyTsetlinMachineParallel (accessed on 24 May 2026). Low hardware and computational footprint, fast training and quick convergence make TM/CTM suitable for on-device learning in many at-the-edge applications such as industrial and environmental sensors, traffic monitors, wearables, smart packaging, etc. The proof-of-concept FPGA and CMOS designs demonstrated the merit of their performance and power efficiency for on-chip online training, which reached 60.3 *k* classifications per second, spending only 8.6 nJ per classification (160 TOPs/J) operating at 27.8 MHz [22,23].

### 2.4. Data Preprocessing: Booleanization and Encoding

**Figure 3:**
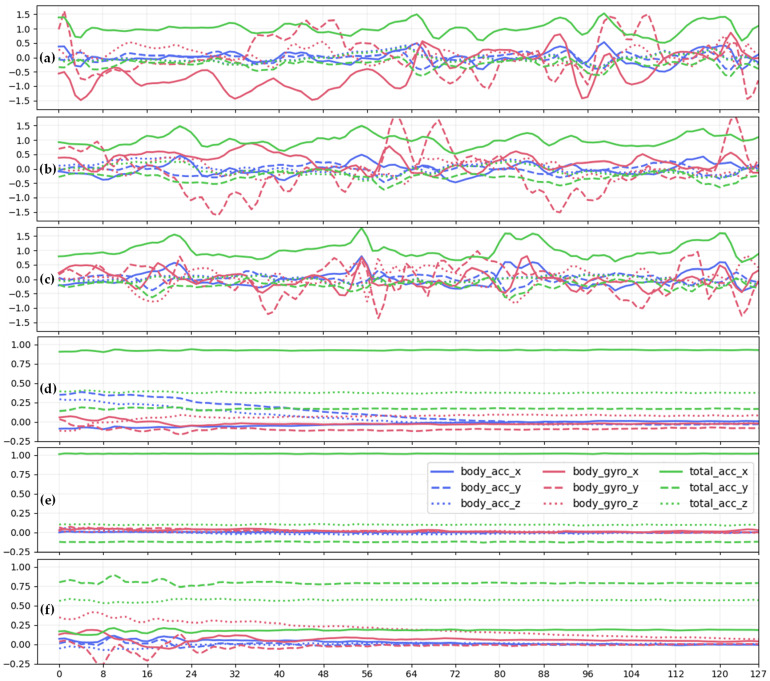
Samples of raw inertial data from the UCI-HAR dataset for different human activities: (**a**) WALKING; (**b**) WALKING_UPSTAIRS; (**c**) WALKING_DOWNSTAIRS; (**d**) SITTING; (**e**) STANDING; (**f**) LAYING. Transient regions are visible near the beginning and end of the 128-sample windows, particularly for the static activities, and the acceleration and gyroscope channels behave distinctly for dynamic versus static activities. Note that a zoomed-in *y*-axis scale is used for the static activities (**d**–**f**) to better reveal the minor fluctuations in the inertial signal values.

**Figure 4:**
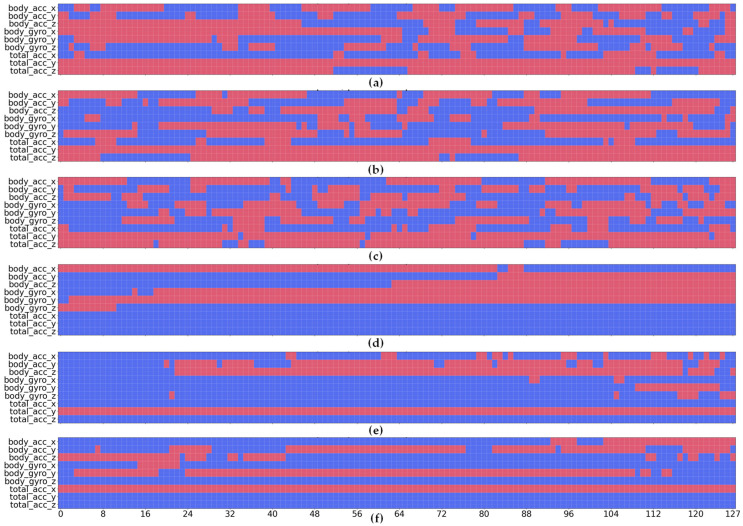
Booleanized inertial signals from the UCI-HAR dataset for the activity samples shown in Figure 3, using the per-channel global mean as a single threshold: (**a**) WALKING; (**b**) WALKING_UPSTAIRS; (**c**) WALKING_DOWNSTAIRS; (**d**) SITTING; (**e**) STANDING; (**f**) LAYING. Blue pixels denote *True* literals, indicating that the corresponding signal value at a given time point is above the channel threshold; red pixels denote *False* literals, meaning that the signal value is equal to or below the threshold.

Tsetlin Machines operate on Boolean input data. Therefore, raw sensor measurements (Figure 3) must first be transformed into Boolean literals, as is visualized in Figure 4. Each literal indicates whether a sensor value satisfies a particular threshold condition or not, i.e., whether it lies above a given channel-specific threshold or within a certain semi-quantitative range. In the simplest setting (B=1), each sensor value is converted into a single Boolean feature using one threshold θ per channel (e.g., mean value computed from the training set). For instance, if the training-set mean of *total_acc_x* is θ=0.55 and a sensor value at a given time point $t_{q}$ is 0.61, the positive literal $total\_acc\_x\left(t_{q}\right)>\theta$ is *True* and is visualized as a blue pixel; if the value is 0.49, the literal is *False* and the negated condition $total\_acc\_x\left(t_{q}\right)\leq \theta$ will be included in the data sample, shown as a red pixel. Booleanization converts continuous sensor signals to a symbolic representation that is suitable for rule-based learning, visualization, and interpretation. However, the granularity of this transformation is important. Too few bins/thresholds may obscure fine-grained variations in accelerometer and gyroscope signals, reducing the model’s ability to distinguish between activities with similar motion patterns but different sensor amplitudes. Conversely, too many bins increase the number of Boolean features and therefore require the TM to allocate more Tsetlin Automata. This increases the memory footprint, computational cost, and training time and can also lead to model overfitting.

The experiments reported in Section 3 therefore illustrate this trade-off between resource efficiency and predictive performance: coarse Booleanization is compact and easier to interpret, whereas finer binning can improve classification accuracy at the cost of a larger and more computationally demanding model.

To Booleanize the inertial signals with multiple bins, we evaluated binning strategies based on equal-width, *k*-means and quantile-based thresholds. For the best-performing quantile strategy, thresholds for each channel i were computed only from the training set as $\theta_{i,b}=Q_{i}\left(b/B\right)$, for i=1,…,N and b=1,…,B, where B is the number of thresholds and $Q_{i}\left(\cdot \right)$ is the quantile function of channel i. These thresholds were then fixed and applied unchanged to both training and test samples S.

Quantile thresholds were preferred because inertial data values are often unevenly distributed and highly concentrated around central near-rest ranges; quantile binning produces more balanced Boolean activations than equal-width intervals.

The resulting semi-quantitative representation was then encoded using thermometer encoding [24], which is particularly suitable for Booleanizing continuous sensor signals because it preserves the ordinal relation between amplitude ranges. This is more robust than one-hot encoding, where adjacent bins are orthogonal, and more interpretable than compact binary coding, where bit positions do not correspond directly to ordered amplitude thresholds. The ordered Boolean vector remains compatible with the clause-based learning mechanism of the TM.

For a channel-specific threshold $\theta_{i,q}$, the b-th thermometer bit is defined as

$$x_{i,q,b}=\left\{\begin{array}{l} 1,\;\;s_{i}\left(t_{q}\right)>\theta_{i,b}\\ 0,\;\;otherwise \end{array}\right..$$

The associated negated literal $\neg x_{i,q,b}$ is also available in a clause.

To illustrate this Booleanization and encoding process with a concrete example, consider the channel *total_acc_x* and a 5-bin quantile Booleanization. Suppose the four 25% quantile thresholds learned from the training data for this channel are $\theta_{total\_acc\_x}=\langle -0.20,\;0.05,\;0.30,\;0.65\rangle$. For a sample value of $total\_acc\_x\left(t_{q}\right)=0.42$ at a given time point $t_{q}$, the value exceeds the 1st, 2nd and 3rd thresholds but not the 4th, so the thermometer-encoded Boolean vector for this channel and time step is 〈1,1,1,0〉. Each bit of this vector forms one positive Boolean literal, e.g., $total_{a}cc_{x}>-0.20$ is *True* (1) and a corresponding negated literal, e.g., $\mathrm{NOT}\left(total\_acc\_x\left(t_{q}\right)>-0.20\right)$ is *False* (0).

Concatenating these vectors over all 9 channels and 128 time steps within a patch produces the input literal vector *L* referred to in Figure 1. A clause $c_{j}^{m}\left(L\right)$ is then a conjunction of a subset of these literals, e.g.,

$$c_{j}^{m}\left(L\right)=\left(total\_acc\_x\left(t_{1}\right)>0.30\right)\;\mathrm{AND}\;\left(\mathrm{NOT}\left(total\_gyro\_z\left(t_{4}\right)>0.10\right)\right)\;\mathrm{AND}\ldots ,$$

which evaluates to *True* only if every included literal in the clause is satisfied by the input sample. During inference, each clause that evaluates to *True* casts one vote for its associated class *m*; the class sum $v_{m}$ is the sum of votes from all clauses associated with class *m* (with clauses assigned a negative polarity contributing negative votes), and the predicted class is the one with the highest class sum (argmax over $v_{m}$), as shown in Figure 1.

### 2.5. Optimization of the Tsetlin Machine Hyperparameters

Compared with deep neural networks, the Tsetlin Machine uses a relatively small set of hyperparameters, as discussed in detail in [17,25]. In this study, we tuned the following main CTM hyperparameters: the number of clauses per class *C*, the voting threshold *T*, the learning sensitivity *s* and the patch length *P*. In addition, we examined the granularity of Booleanization; although it is not a hyperparameter of the TM itself, it directly affects the dimensionality and informativeness of the Boolean input representation and, therefore, has a substantial impact on performance. The objective was to maximize classification performance and to understand how model complexity, Boolean feature granularity, memory footprint and computational cost affect the suitability of the CTM for resource-constrained HAR.

The number of clauses *C* defines TM’s learning potential—the more clauses, the more variations in class patterns the TM can learn, generally improving classification performance up to a saturation point [25]. However, using more clauses increases computational cost and energy consumption and results in a larger memory footprint and longer training. For the base CTM model, we used 100 clauses per class (*C* = 100), which offers a practical compromise between representational capacity and model size and is a common default in many examples of TM application [21].

The size of the TM model itself is determined by the number of used Tsetlin Automata. This number is proportional to the number of classes, the number of clauses per class and twice the number of Boolean input features, since each feature is represented by both a positive and a negated literal. With one Boolean threshold applied to each of the 128 samples from nine inertial channels, the input contains 9 × 128 Boolean features. For six HAR classes and 100 clauses per class, this gives 1,382,400 TAs. Taking that each TA by default occupies one byte of memory to store its 256 states (2n=256), the resulting model size is therefore 1350 KB. This illustrates the compactness of the CTM compared with many deep learning models, as discussed in Section 3.1, especially considering that the model performs multiclass activity recognition directly from raw inertial data without relying on float pointing numbers and the costly gradient descent technique.

Moreover, once training is complete, deploying the TM for inference requires storing only the final include/exclude actions of each TA, rather than its full internal state. Since each learned TA action can be represented by a single bit, with 0 denoting exclusion and 1 denoting inclusion of the corresponding literal in a clause, the inference-only model will require as little as 169 KB of memory. This footprint can be compressed even further using the approach proposed in [26].

The voting threshold *T* is another important hyperparameter controlling the confidence level of the vote-based inference of the TM. We set it following the heuristic that the optimal *T* scales with the square root of half the number of clauses [25]; this heuristic has been shown to align with the *Jagiellonian compromise* [27], which defines the optimal quota for a qualified majority in Penrose’s square-root voting system [28]. Thus, for the base configuration of 100 clauses per class, we tested voting thresholds in the range 7 ± 3, with *T* = 9 giving the best accuracy; adding 1–2 votes to the heuristic value $\sqrt{C/2}$ generally increases TM performance and stability due to higher voting confidence [25].

The learning sensitivity *s*, which regulates the balance between clause generalization and specialization [29], was determined experimentally by varying its value between 1.5 and $\left(C-2T\right)/T$ as recommended in [25,29], in steps of 0.25. The optimal value in our experiments was approximated to 3.75, providing a balanced trade-off between learning broadly reusable activity patterns and forming sufficiently specific and discriminative clauses that minimize false-positive predictions. The patch size was varied over 9 × {1, …, 128} and the number of Booleanization thresholds over the range {1, …, 10}.

The best hyperparameter configuration achieving the highest validation accuracy after 1000 training epochs (Table 1) was selected from the grid search using the two-stage approach. In the first stage, we identified the values of *T* and *s* that provided the best validation performance (the validation subset comprised 20% of the training set using a subject-disjoint split) for the full 9 × 128 patch using a single Booleanization threshold. In the second stage, we fixed these parameters and investigated the effect of patch size and Booleanization granularity. This two-stage decomposition was adopted both to keep the search tractable and because *T* and *s* are the primary hyperparameters governing the TM’s learning capability—in our experience, a TM with well-chosen (*T*, *s*) consistently outperforms otherwise-identical TMs that differ only in secondary settings such as the patch size or the data-encoding scheme. Optimizing the primary hyperparameters first therefore provides a strong, stable basis on which the secondary settings can subsequently be tuned.

The CTMs with the best performing configurations of hyperparameters were then evaluated on the test set, with results averaged over five independent training runs, as reported in Section 3.1. This protocol ensures that the reported test accuracies are not directly optimized on the test data: because UCI-HAR provides a fixed, published subject-wise train/test split, the test set was used only once for final reporting per selected configuration.

## 3. Results

### 3.1. Performance of the Convolutional Tsetlin Machine

This section reports the performance of the Convolutional Tsetlin Machine on the UCI-HAR dataset using raw accelerometer and gyroscope signals. The stochastic reinforcement mechanism intrinsic to TM learning causes it to exhibit slightly different performance across independent training runs, even when identical hyperparameters are used. This run-to-run variation does not, however, correspond to statistically distinct models. The statistical equivalence of independently trained equivalent TMs has been confirmed in [17], where two-sample *T*-, *F*-, and *Kolmogorov*–*Smirnov* tests indicated with high confidence that their learning results share the same mean, variance and underlying distribution. Consequently, averaging performance over several independent runs is statistically justified. It is also practically beneficial to retain the best-performing TM across these runs. As such, Table 2 reports TM performance as a function of patch size and Booleanization granularity after 1000 training epochs, averaged over five training runs, while the best achieved performance is shown in Table 3, where it is also compared with other ML methods run in the same environment. To provide a more detailed, per-activity view beyond the aggregate metrics, Table 4 presents the confusion matrix for the best-performing CTM, which reveals how errors are distributed across the six activities and identifies which classes are most frequently confused with one another.

In our first round of experiments, each sensor value was converted into one Boolean feature using a single threshold. For the base CTM model (*C* = 100, *T* = 9, *s* = 3.75), the patch size was set to be equal to the full 9 × 128 input window, allowing each clause to observe the complete temporal sequence across all nine inertial channels at once. The full-patch model provided a reasonably strong baseline, achieving a test accuracy of 86.51% on average ± 0.14% after 1000 training epochs (Table 2). This result confirms that the CTM can learn meaningful HAR patterns from raw Booleanized inertial signals even without handcrafted feature extraction, and that even a very compact Boolean representation can support effective HAR classification.

Further experiments showed that reducing the temporal patch length substantially improved classification accuracy. The CTM with as few as 100 clauses per class reached peak accuracy above 91% for several shorter patch sizes, including 9 × 114, 9 × 100, 9 × 89 and 9 × 70. Two complementary effects likely explain why a moderately reduced patch size improves performance. For dynamic activities, the repeatable gait patterns may begin at slightly different phases in each 128-sample window. Thus, a smaller patch, sliding across the window, can localize the same pattern regardless of its exact position, giving the CTM greater flexibility and improving generalization. For some static activities, the beginning and end of each window may instead contain less informative transient regions, as can be seen in Figure 3 and Figure 4; here, a moderately reduced patch allows the CTM to focus on the more stable central activity signatures. A smaller patch size also has a direct impact on model size, proportionally reducing the number of used TAs. However, the reduction in memory footprint comes with computational overheads as smaller patches increase the number of candidate patch positions that must be evaluated within each input window, hence increasing both training time and inference latency. Table 2 shows this trade-off for the 9 × 114 patch size compared with the base full-window configuration.

Finally, we investigated the effect of increasing Booleanization granularity on CTM performance, as a single threshold could be too coarse to capture finer amplitude differences in the inertial signals, especially for activities with similar motion or posture patterns. More granular Booleanization was therefore evaluated using multiple thresholds. Among the tested approaches, quantile-based thresholds provided better results as compared to equal-width binning and *K*-means binning.

The best overall performance was obtained with five bins using four quantile thresholds and applying the thermometer encoding, where the CTM achieved 94.92 ± 0.31% test accuracy after 1000 epochs, while the training accuracy reached 99.58 ± 0.05%. This configuration provided the most balanced class-level performance for which the dynamic activities were classified with high accuracy: WALKING reached an accuracy of 97.06 ± 1.07%, WALKING_UPSTAIRS—94.35 ± 0.96% and WALKING_DOWNSTAIRS—99.47 ± 0.39%. The LAYING class was perfectly recognized with 100% accuracy. The most important improvement was observed for the more difficult postural classes: SITTING reached an accuracy of 88.15 ± 1.43%, while STANDING reached 90.94 ± 0.84%, indicating that additional Boolean thresholds help the CTM distinguish between activities with similar static inertial signatures. Further increasing the number of bins did not provide a consistent improvement in test accuracy; instead, it increased the risk of CTM overfitting and added memory and computational overhead. Table 2 shows that both applying a sliding window and increasing the granularity of Booleanization independently improve the overall accuracy of the CTM. The per-class results, however, show that different activities benefit from different mechanisms. SITTING, the hardest class to recognize, gains substantially from finer Booleanization, raising from 63.75% to 82.85% on average at the full patch when moving from two to five bins. Thus, additional thresholds help resolve the subtle signal differences between similar static postures. WALKING and WALKING_UPSTAIRS, by contrast, benefit more from the sliding window: WALKING rises from around 89–90% to 97–98% and WALKING_UPSTAIRS from roughly 86% to 94% once the patch size is reduced, suggesting that the convolutional sliding window better captures the repetitive temporal patterns of dynamic activities. LAYING is already perfectly classified under all configurations and is therefore unaffected by either change. These gains come at the cost of increasing the model size and slowing both training and inference. Thus, the accuracy improvements must be weighed against efficiency when targeting resource-constrained deployment.

As shown in Table 3, the best-performing CTM configuration using a 9 × 114 patch and five-bin Booleanization achieves a test accuracy of 95.11%, outperforming classical and deep-learning baselines trained and evaluated on the raw inertial signals under the same experimental conditions while keeping a compact 601 KB deployment footprint. This accuracy comes with good computational efficiency—the best CTM configuration required 1859 s for training and delivered an inference throughput of 4886 ops/s.

Although slower than the classical models, such as Random Forest, the CTM trains faster than the main deep-learning competitors and provides substantially higher inference throughput in terms of predictions per second. Several recently proposed methods report higher accuracy on the raw inertial signals of UCI-HAR, such as the homogeneous stacked TCN ensemble with a Nu-SVC meta-learner (97.25% [34]) and a ResNet trained with channel-selective convolutions (97.28% [35]). However, they rely on complex multi-model ensembles and/or prior manipulation of the inertial channels (e.g., channel deallocation and reallocation), and proposed methods lack sufficient implementation details for faithful reproduction; they were therefore not included as direct baselines in Table 3, but are noted as representative of the current upper end of reported accuracy on this dataset.

### 3.2. Decision Making and Clause Visualization of the Convolutional Tsetlin Machine

**Figure 5:**
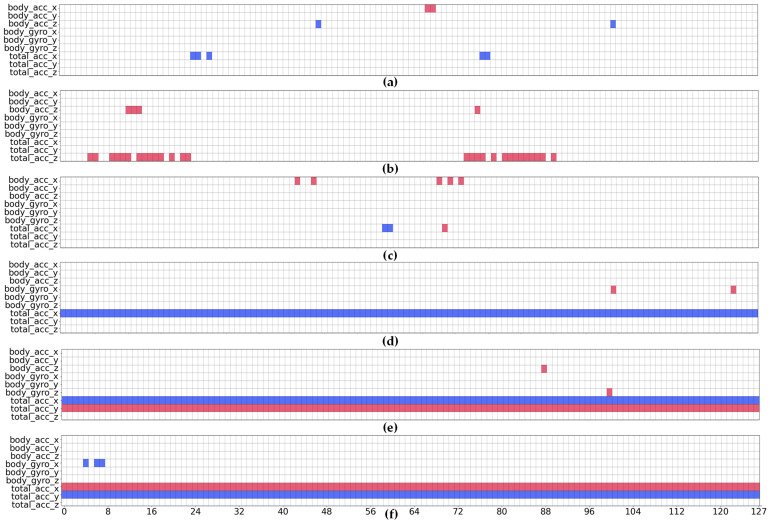
Example of individual clauses after training the base CTM model with one threshold per channel and a full 9 × 128 patch size for different activities: (**a**) WALKING; (**b**) WALKING_UPSTAIRS; (**c**) WALKING_DOWNSTAIRS; (**d**) SITTING; (**e**) STANDING; (**f**) LAYING. A blue pixel means that the value of the corresponding signal in the corresponding position must be above the threshold for that channel to match the clause rule. Red means that the signal value must be equal to or below the threshold. Finally, white means ‘ambivalent’, i.e., the signal value *may* be either above or below the threshold.

The Tsetlin Machine performs classification through the construction and evaluation of conjunctive logic statements, referred to as clauses. During training, each clause learns to include or exclude selected Booleanized input features and their negations. Each CTM’s clause therefore represents a logical pattern over the inertial signal window. If all selected literals in the clause are satisfied by an input sample, the clause evaluates to *True* (1) and contributes a vote towards a class. If one or more selected literals are not satisfied, the clause evaluates to *False* and abstains from voting. In the HAR setting, each Boolean feature corresponds to a thresholded inertial signal value at a specific sensor channel in a certain temporal position. Figure 5 visualizes examples of learned CTM clauses after training using one threshold per channel. In these visualizations, a blue pixel indicates that the corresponding positive literal is included in the clause, meaning that the inertial signal value at that channel and time position is required to be above the threshold. A red pixel indicates that the corresponding negated literal is included, meaning that the signal value is required to be below or equal to the threshold. A white pixel means that the literal is excluded from the clause and, therefore, its value does not affect whether the clause activates. This visual representation makes the internal structure of the CTM directly interpretable. Each clause can be read as a sparse logical stencil over the sensor window, specifying which signal conditions are relevant for recognizing a particular activity. For example, a clause may require selected acceleration values in one channel to be above the threshold while simultaneously requiring values in another channel to remain below the threshold. Such combinations allow the CTM to capture activity-specific motion signatures, including both the presence and absence of particular inertial patterns. During inference, a data sample is matched against all clauses associated with all classes. If it satisfies every included literal of a clause, the clause outputs *True* and casts a vote for its designated class. Otherwise, the clause outputs *False* and does not contribute to the class score.

The final prediction is obtained by aggregating the clause votes and selecting the class with the highest total vote. This means that the predicted activity is not determined by a single rule, but by the collective evidence accumulated from many independently learned clauses. Such a clause-voting mechanism provides a form of collaborative decision-making. Different clauses may capture different local temporal fragments, sensor channels, or motion characteristics of the same activity. Some clauses may represent strong but specific patterns, while others may encode more general conditions that occur across a wider range of samples. The final class decision is therefore based on the combined support of multiple logical patterns rather than on one monolithic model response. This is particularly useful for HAR, where the same activity can vary across users, repetitions, and sensor placements.

### 3.3. Tsetlin Machine Interpretability and Visualization of Human Activity Patterns

**Figure 6:**
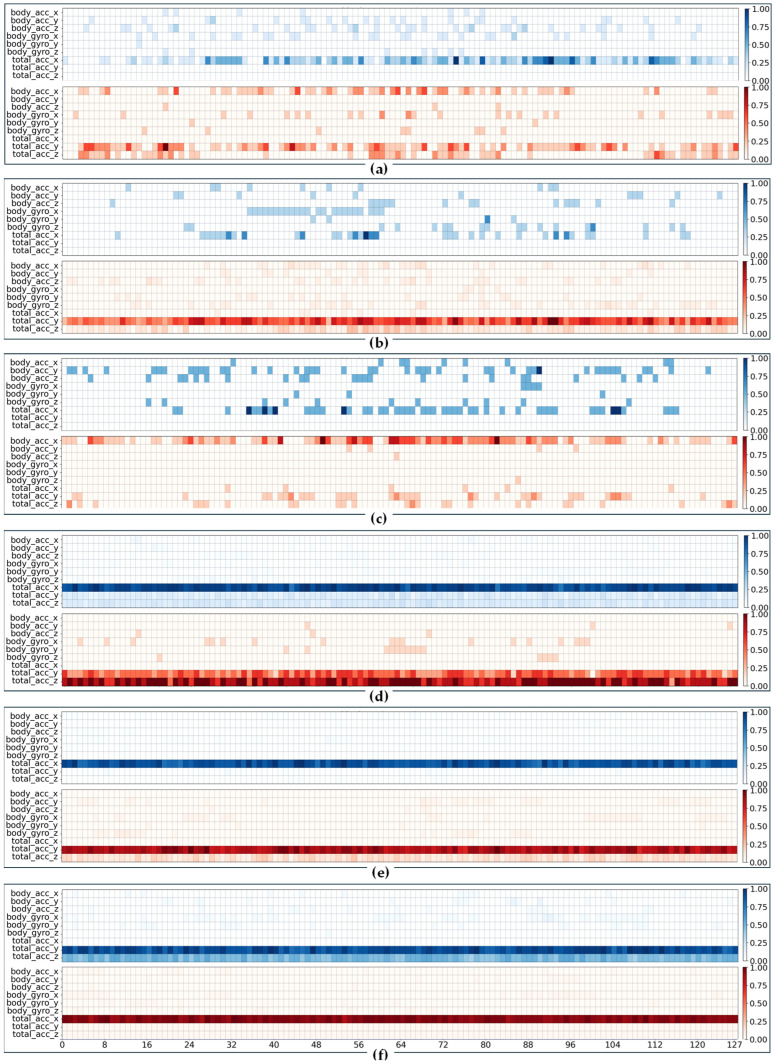
Class patterns obtained by aggregating together clauses of the base CTM model with one threshold per channel for different activities (positive ‘blue’ and negated ‘red’ literals were grouped separately): (**a**) WALKING; (**b**) WALKING_UPSTAIRS; (**c**) WALKING_DOWNSTAIRS; (**d**) SITTING; (**e**) STANDING; (**f**) LAYING. The more saturated the blue or red color, the more often the corresponding signal or its negated value occurs in the corresponding position across clauses of the class.

Collectively, the clauses associated with each class form an aggregated class template that highlights the most discriminative Boolean features for that activity. Figure 6 presents these templates by grouping positive (blue) and negated (red) literals separately, providing insight into how the CTM exploits Booleanized inertial signals to distinguish between different HAR classes. Blue, red and white regions have the same meaning as explained in Section 3.2 for Figure 5. The more saturated the blue or red color, the more often the corresponding signal or its negated value occurs in a certain position across clauses of the class. Overall, the templates show that the CTM does not use all channels uniformly. Instead, it selects sparse and class-specific combinations of literals. This confirms that the learned clauses act as interpretable activity templates: they identify which sensor channels and temporal regions are most useful for recognizing each activity. The strongest and most consistent patterns appear in the total acceleration channels, especially *total_acc_x* and *total_acc_y*, while gyroscope channels are used more selectively, mainly contributing to specific dynamic activities:

- Class 0 (WALKING): The class template shows strong use of *total_acc_x* across many temporal positions, with additional but weaker contributions from *body_acc_x*, *body_acc_y*, *body_acc_z* and some gyroscope channels. This indicates that walking is recognized through a distributed acceleration pattern rather than through a single isolated time point. The template also includes many negated activations, almost uniformly spread across *body_acc_x*, *total_acc_y*, *total_acc_z* and much less frequent but regular activations in *body_gyro_x*, showing that walking is characterized not only by the presence of above-threshold acceleration in some channels, but also by the absence of above-threshold values in others. This combination of positive and negated literals is consistent with a *periodic locomotion pattern*, where the model learns both high and low phases of the motion cycle.
- Class 1 (WALKING_UPSTAIRS): The most striking pattern is the strong and continuous negative-literal band in *total_acc_y*. This means that many clauses for this class require *total_acc_y* to remain below the threshold across much of the temporal window. At the same time, the template contains localized blue regions in *body_gyro_x*, *body_gyro_z* and *total_acc_x*. This suggests that upstairs walking is distinguished by a combination of constrained vertical or orientation-related acceleration behavior and specific rotational components. Compared with ordinary walking, the class relies less on broadly distributed positive acceleration and more on a stable below-threshold condition in the dominant *total_acc_y* acceleration channel.
- Class 2 (WALKING_DOWNSTAIRS): The positive literals are more distributed across *body_acc_y* and *total_acc_x*. The template also shows strong negated activations in *body_acc_x* together with much less frequent but regular negative evidence in *total_acc_y* and *total_acc_z*. This suggests that the CTM distinguishes downstairs walking through both above-threshold motion bursts and below-threshold constraints in acceleration channels, primarily in *body_acc_x* as opposed to *total_acc_y*, which is more characteristic for WALKING_UPSTAIRS.
- Class 3 (SITTING): The template is highly structured and much less sparse than the dynamic classes. The template is dominated by an almost continuous blue band in *total_acc_x*, a strong continuous red band in *total_acc_z* and *total_acc_y*, as well as sporadic but regular negated values in *body_gyro_x*. This means that many clauses describe sitting using a stable relation between two total acceleration components: *total_acc_x* being above the threshold and *total_acc_z* and *total_acc_y* being below the threshold. Such a pattern is consistent with a static posture, where the discriminative information is not rapid temporal variation but the orientation of the body and device relative to gravity. The near-continuous horizontal bands in certain channels show a persistent posture signature across the full window, which is common for all static Classes 3–5.
- Class 4 (STANDING): The clause template is quite similar to Class 3. It also contains a strong positive band in *total_acc_x* and a strong negative band in *total_acc_y*. This explains why sitting and standing are the most frequently confused classes. Both activities are static and are represented by similar long-duration acceleration-orientation conditions. However, the standing template appears even cleaner and more uniform, with fewer additional selected literals in the other channels. This suggests that standing is represented by a more stable inertial configuration, while sitting may require slightly more auxiliary evidence. The similarity between Classes 3 and 4 confirms that the CTM’s classification difficulty shown in the confusion matrix (Table 4) is not random: it arises from genuinely overlapping Boolean signal templates.
- Class 5 (LAYING): The template is the most distinctive among all classes. It contains a strong continuous blue (i.e., positive) band in *total_acc_y* and an additional weaker but still persistent blue band in *total_acc_z*. It also contains a very strong continuous red (i.e., negated) band in *total_acc_x*. This indicates that laying is recognized through a stable acceleration-orientation pattern combined with a strong absence of rotational movement. This is consistent with the classification results (Table 4), where laying achieves perfect precision and recall. Unlike sitting and standing, laying produces a more distinctive orientation of the device relative to gravity, making it much easier for the CTM to separate from other classes.

A key observation is that dynamic activities (WALKING, WALKING_UPSTAIRS and WALKING_DOWNSTAIRS) are represented by more fragmented and distributed clause templates. Their discriminative literals occur in multiple channels and at multiple temporal positions, reflecting local motion events and temporal variability. In contrast, static activities (SITTING, STANDING and LAYING) are characterized by long continuous horizontal bands, especially in total acceleration channels. This indicates that the CTM uses persistent above-threshold or below-threshold conditions as posture templates.

### 3.4. Sensor and Channel Contribution Analysis

Existing research, e.g., [5,6,36,37], has shown that ML-based human activity recognition can benefit substantially from multimodal sensor fusion by combining accelerometer, gyroscope, magnetometer and other wearable signals, which provide more discriminative movement representations than a single sensor modality alone. However, the individual contribution of each sensor, signal component or axis is less frequently analyzed in detail, while it is widely accepted that different activities trigger different physical cues, e.g., dynamic locomotion classes usually rely on acceleration and angular-velocity patterns, whereas static postures may be distinguished mainly through orientation-related acceleration signals. Our experiments confirm that the contribution of each modality is unequal (Table 5) and highly activity-dependent (Table 6). Some activities benefit from the full combination of accelerometer and gyroscope channels, while others can be recognized accurately using a smaller subset of signals. This suggests that sensor selection should not be treated only as a fixed preprocessing choice, but as an important design parameter for resource-aware HAR.

**Figure 7:**
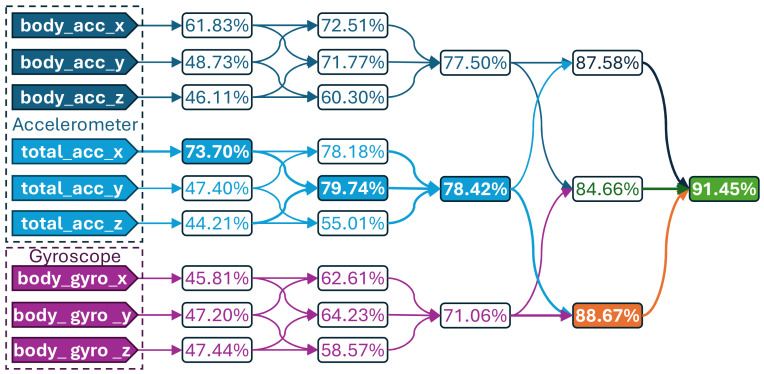
Contribution of different inertial signals and their combinations into HAR performance for the CTM (*C* = 100, *T* = 9, *s* = 3.75) using a 9 × 114 patch size and a single Booleanization threshold.

The CTM with 9 × 114 patch size and a single Booleanization threshold achieved the best overall result, reaching 91.45% test accuracy, when all nine channels were used. However, the six-channel combination of total acceleration and gyroscope already achieves 88.67%, indicating that much of the useful discriminative information is contained in these two modalities. In contrast, the combination of body acceleration and gyroscope reaches 84.66%, while body acceleration and total acceleration reaches 87.58% (Figure 7). This suggests that total acceleration carries particularly strong information for this dataset, especially because it combines body-motion and gravity-related components. Among single channels, *total_acc_x* is by far the strongest, achieving 73.70% test accuracy. This is substantially higher than *body_acc_x* at 61.83%, while all other single channels are below 49%.

Therefore, *total_acc_x* is the most important individual channel overall for recognizing six activities from the UCI-HAR dataset. Among complete three-axis sensors, *total_acc_xyz* is strongest with 78.42%, followed by *body_acc_xyz* with 77.50%, and *body_gyro_xyz* with 71.06%. Thus, the total acceleration sensor is the most informative single sensor overall, although the gyroscope becomes highly valuable when fused with total acceleration. The activity-wise performance analysis (Table 6 reports activity-wise F1-scores) showed a clear separation between dynamic and postural activities:

- Class 0 (WALKING): The strongest single-channel result is *total_acc_x*, with a recall of 0.96 and F1-score of 0.86. *Body_acc_x* also performs well with a recall of 0.88 and F1-score of 0.80. This implies that walking is strongly characterized by forward-axis acceleration patterns and can be recognized effectively with a low-cost single acceleration channel, particularly *total_acc_x*. Gyroscope-only channels are less useful for walking, although *body_gyro_x* still gives a relatively high recall of 0.89 and an F1 score of 0.81.
- Class 1 (WALKING_UPSTAIRS): No single channel is sufficient for robust recognition (the *total_acc_x* classifier obtains an F1-score of 0.73, while *body_acc_y* gives 0.68 at maximum). Performance improves substantially when multiple axes are combined: *body_acc_xyz* and *body_gyro_xyz* both give an F1-score of 0.86, and the full nine-channel model achieves an F1-score of 0.97. The confusion matrices showed that Class 1 is often confused with classes 0 and 2 if only one channel is used. This is expected because walking upstairs shares a periodic gait structure with both level walking and walking downstairs. Therefore, Class 1 requires multi-axis information, preferably including acceleration and gyroscope signals.
- Class 2 (WALKING_DOWNSTAIRS): The *x* and *z* body-acceleration axes are especially informative for downward locomotion, by capturing vertical/forward movement components. The *body_acc_xz* model correctly classified 412 out of 420 WALKING_DOWNSTAIRS samples, ensuring a recall of 0.98 and F1-scores of 0.95, similar to the whole *body_acc_xyz* (0.96). The gyroscope channels also contributed, but less strongly: the F1-score for *body_gyro_xyz* was 0.80 at maximum.
- Class 3 (SITTING): It is the most challenging activity to recognize, often confused with STANDING. For example, *total_acc_x* alone almost completely failed for Class 3 (only 1 out of 491 sitting samples was correctly recognized, while 472 were misclassified as Class 4). This means *total_acc_x* is practically useless for recognizing sitting, despite being the best single channel overall. Similarly, *total_acc_z* gave a zero F1 score for this class, while *body_acc_x* alone was also weak, with F1 only reaching 0.26. The best results for Class 3 appeared only after multimodal fusion: the full nine-channel model reached an F1-score of 0.79, and *total_acc_xyz_body_gyro_xyz* ensured F1-score at the level of 0.75.
- Class 4 (STANDING): For this class, the total acceleration is highly informative. *Total_acc_x* alone achieves 1.00 recall for Class 4, but this comes at the cost of misclassifying almost all sitting samples as standing, producing only 0.53 precision. The best balance is obtained from fused models. The full nine-channel input achieved an F1-score of 0.86, while *total_acc_xyz_body_gyro_xyz*—0.85 and *body_acc_xyz_total_acc_xyz*—0.81, respectively. The gyroscope helped reduce confusion between sitting and standing, although even the best model still misclassified some sitting samples as standing;
- Class 5 (LAYING): *total_acc_x* alone correctly classified all 537 test samples of this class, giving a recall of 1.00 and an F1-score of 0.99. Other total acceleration channels, such as *total_acc_y* and *total_acc_z*, also performed well, with recalls being at the level of 0.90 and 0.91 and F1 scores of 0.73 and 0.69, respectively. This indicates that laying is strongly defined by gravitational orientation rather than dynamic motion. Gyroscope-only channels are much weaker for laying, especially *body_gyro_x,* with an F1-score of 0.33. Therefore, gyroscope channels are practically unnecessary for recognizing laying if total acceleration is available.

The least useful individual channels overall are *total_acc_z*, *body_gyro_x*, *body_acc_z*, *body_gyro_y*, *total_acc_y* and *body_gyro_z*, all producing the average test accuracies around 44–47%. However, their usefulness is activity-specific. For example, *total_acc_z* is poor overall and completely failed to recognize sitting, but it still helped recognize laying. *Body_gyro_x* was weak as a single channel but became useful in gyroscope combinations, especially *body_gyro_xz*, which achieved 64.23% accuracy, and *body_gyro_xyz*, which reached 71.06% overall. This indicates that gyroscope axes are not individually strong, but their rotational information becomes valuable in combination.

From an energy-efficiency perspective, the results suggest several practical trade-offs. If minimum sensing cost is required, *total_acc_x* is the best single-channel configuration, achieving 73.68% accuracy and 67.68% F1-score with only one channel. However, this setting is unsuitable when sitting must be recognized. If a moderate-cost configuration is acceptable, *total_acc_xz* is highly attractive: it uses only two channels but reaches 79.74% accuracy and 79.33 F1, outperforming all three-axis single-sensor configurations. If high accuracy is required, *total_acc_xyz_body_gyro_xyz* provides 88.66% using six channels, only 2.79 percentage points below the full nine-channel model. This suggests that body acceleration may be partly redundant when total acceleration and gyroscope signals are available.

## 4. Discussion

In the paper, we demonstrated that the Convolutional Tsetlin Machine can achieve strong HAR performance on raw inertial data while retaining a compact and interpretable clause-based structure with low memory and computation footprint. UCI-HAR dataset used in the study offers a useful compromise between benchmark popularity, technical clarity, raw-signal availability, and computational manageability. While larger or more sensor-rich datasets such as PAMAP2, WISDM, or OPPORTUNITY are valuable for broader multimodal evaluation, UCI-HAR is especially suitable as an initial benchmark for analyzing CTM-based raw-signal learning, Booleanization strategies, patch-size selection, clause interpretability and sensor-modality-aware classification.

The base full-patch model confirmed the feasibility of CTM-based HAR, while the optimized configurations showed that accuracy could be substantially improved by selecting an appropriate patch size and Booleanization granularity. The best configuration, using reduced temporal patches (e.g., 9 × 114) and five-bin quantile Booleanization, provided a favorable balance between classification accuracy, memory footprint and computational cost, reaching 95.11% accuracy after 1000 training epochs. These findings support the suitability of CTM for resource-aware on-device HAR for inference and potentially for on-device learning, where predictive performance, efficient model representation and explainable decision making are essential. CTM-based HAR considerably differs from conventional black-box learning approaches. The model not only outputs a predicted activity label but also provides a set of inspectable logical clauses that explain how this prediction is formed, and exposes the sensor channels and temporal regions used in its decision process. This native interpretability is especially important for wearable HAR applications in healthcare, rehabilitation, sport and safety monitoring, where understanding why an activity or anomaly is detected may be as important as the prediction itself.

The learned CTM clauses reveal that locomotion (walking and stair climbing) is captured well by the body-acceleration and gyroscope channels, while total acceleration is the strongest single contributor for laying, encoding the device orientation that distinguishes it. The static postures, sitting and standing, are the most demanding, resolved only by the full multimodal combination of acceleration and gyroscope signals.

Regarding channel modalities, *total_acc_x* is by far the strongest single inertial channel that achieved 76.68% of train and 73.70% of test accuracy overall. It is also excellent at detecting a broad ‘upright/static’ state, but largely fails at separating sitting from standing. Body acceleration, especially the *x* and *z* axes, is highly important for identifying locomotion patterns. The *y*-axis alone is weaker, suggesting that lateral or vertical components are less discriminative for UCI-HAR activities when used in isolation. Sitting activity requires complementary orientation and rotational information obtained from all nine channels and cannot be reliably recognized from a single acceleration axis.

By identifying which channels are most informative for particular activity classes, it becomes possible to reduce sensing, memory and computational requirements by discarding signals with limited discriminative value while maintaining competitive classification accuracy. In wearable systems, gyroscopes typically consume more energy than accelerometers. Therefore, an accelerometer-only configuration is preferable for low-power HAR when the target activities are mainly walking, walking upstairs, walking downstairs and laying. However, gyroscope information is valuable for resolving difficult postural ambiguities, especially sitting versus standing. The best engineering compromise is therefore application-dependent: *total_acc_x* and *total_acc_xz* offer strong low-energy performance, *total_acc_xyz* ensures balanced accelerometer-only recognition, while *total_acc_xyz_body_gyro_xyz* allows to achieve near-full accuracy with fewer channels than the complete nine-channel setup.

The full nine-channel model remains the most accurate, but its additional gain over the six-channel *total acceleration* + *gyroscope*’s configuration is relatively small compared with the likely increase in sensing, memory and processing cost.

## 5. Conclusions

This study evaluated the Convolutional Tsetlin Machine for sensor-modality-aware HAR on the raw inertial signals. The results show that the CTM can achieve competitive accuracy while relying on automata-based learning and clause-based inference, the decisions of which are directly inspectable through clause analysis and visualization.

The systematic modality and channel analysis showed that the discriminative value of each inertial signal is highly activity-dependent: total acceleration, especially *total_acc_x*, is the strongest single contributor to overall accuracy and to the recognition of dynamic activities and laying, while the postural classes require multimodal fusion across acceleration and gyroscope channels. The aggregated clause templates further demonstrated that the CTM’s learned logic-based rules are directly interpretable, exposing which sensor channels and temporal regions drive each classification decision and providing a transparent link between model behavior and the underlying physical motion patterns. However, because all experiments used the waist-mounted smartphone configuration of UCI-HAR, channel-importance findings should be regarded as specific to this sensor placement and dataset until further verified on other HAR benchmarks, such as WISDM, PAMAP2, and OPPORTUNITY. Taken together, these findings support the CTM as a compact, transparent and computationally efficient alternative to conventional deep-learning HAR models, particularly for resource-constrained wearable and edge platforms where on-device learning, low memory footprint and explainable decision making are required. Future work will extend this analysis to additional HAR benchmarks and sensor placements, validate CTM deployment on representative edge and wearable devices and further investigate how learned clause structures can support continual, personalized on-device adaptation.

## Figures and Tables

**Table 1:** CTM hyperparameters, tested ranges, optimal values and other experimental settings.

Hyperparameter	Tested Range	Optimal Value
Number of clauses per class, *C*	100	-
Voting threshold, *T*	$\left\{\sqrt{C/2}-3,\ldots ,\sqrt{C/2}+3\right\}$, step 1	9
Learning sensitivity, *s*	$\left\{1.5,\ldots ,\left(C-2T\right)/T\right\}$, step 0.25	3.75
Patch size, *p*	9 × {1, …, 128}, step 1	9 × 114
Booleanizanion thresholds, *B*	$\left\{1,\ldots ,10\right\}$, step 1	4
Binning approach:		
one threshold	{*mean*, *median*}	*mean*
multiple thresholds	{*equal-width*, *quantile*, *K-means*}	*quantile*
Encoding	{*thermometer*, *one-hot*}	*thermometer*
Training epochs	1000	-
Train/test split	UCI-HAR defined	-
Train/validation split	80/20; subject-disjoint	-

**Table 2:** CTM (*C* = 100, *T* = 9, *s* = 3.75) performance depending on the patch size and Booleanization granularity, averaged over 5 independent training runs.

Performance Metrics	Patch Size 9 × 128		Patch Size 9 × 114	
	2 Bins ^1^	5 Bins	2 Bins	5 Bins
Train accuracy, %	92.59 ± 0.27	98.90 ± 0.25	95.20 ± 0.30	99.58 ± 0.05
Test accuracy, %	86.51 ± 0.14	90.18 ± 0.32	91.03 ± 0.36	94.92 ± 0.31
WALKING	89.07 ± 2.11	90.24 ± 0.97	88.10 ± 2.40	97.06 ± 1.07
WALKING_UPSTAIRS	86.58 ± 2.26	85.18 ± 1.12	94.31 ± 1.40	94.35 ± 0.96
WALKING_DOWNSTAIRS	86.14 ± 1.82	91.71 ± 2.32	99.29 ± 0.29	99.47 ± 0.39
SITTING	63.75 ± 2.81	82.85 ± 2.63	76.17 ± 2.47	88.15 ± 1.43
STANDING	91.28 ± 0.99	90.19 ± 1.34	89.02 ± 1.37	90.94 ± 0.84
LAYING	100.00 ± 0.00	100.00 ± 0.00	100.00 ± 0.00	100.00 ± 0.00
Avg. training time (1000 epochs) ^2^, s	153/575	344/1477	475/776	1844/2196
Avg. inference throughput ^2^, ops/s	106,625/7704	32,581/2660	17,888/1826	5056/608
CTM train model size, KB	1350	5400	1202	4809
CTM deployment size, KB	169	675	150	601

^1^ Base model; ^2^ CTM performance achieved on: i7-12700H CPU with 16 GB RAM/Quad-core Cortex-A53 CPU with 4 GB RAM when deployed on Arduino UNO Q.

**Table 3:** Comparison of the best-performing CTM with other established ML models, all trained and evaluated in the same environment ^1^ on the raw inertial signals of the UCI-HAR dataset.

Model	Reported Accuracy ^2^	Evaluated Performance				Train Time ^3^, s	Throughput, ops/s	Size, KB
		*Acc.*	*Prec.*	*Rec.*	*F*1			
CTM (*C* = 100, *T* = 9, *s* = 3.75) ^4^		0.951	0.951	0.952	0.951	1859	4886	4809/ 601 ^5^
Random Forest [30]	0.910 ^6^	0.850	0.850	0.849	0.848	1	63,565	8487
SVM Classifier [31]	0.946	0.901	0.903	0.902	0.901	6	503	32,176
Stacked LSTM [32]	0.937	0.927	0.931	0.927	0.927	8530	178	702
1D-CNN_Gopinath [30]	0.961 ^6^	0.900	0.903	0.903	0.901	205	1526	1298
1D-CNN_Ronao [31]	0.948	0.943	0.944	0.943	0.942	2019	872	17,306
2D-CNN [33]	0.919	0.935	0.937	0.936	0.934	517	1050	317
RCN [33]	0.937	0.931	0.932	0.931	0.931	3522	330	12,904

^1^ Experiments were performed on an Intel Core i7-12700H CPU with 16 GB RAM; ^2^ Accuracy as reported in the original paper; minor discrepancies may be due to unreported implementation details, such as batch size or training protocol; ^3^ Training time was measured over 1000 training epochs, except for single-step optimization models such as SVM and Random Forest; ^4^ Best performing CTM configuration: 100 clauses per class, a (9 × 114) patch size and 5-bin Booleanization; ^5^ CTM model size: trainable/deployable; ^6^ Accuracy was achieved on the full UCI-HAR dataset, including both raw inertial signals and the 561 preprocessed features.

**Table 4:** Test-set confusion matrix of the best-performing CTM across five runs (95.11% accuracy), using hyperparameters *C* = 100, *T* = 9, *s* = 3.75, a 9 × 114 patch size and 5-bin Booleanization.

Predicted	0	1	2	3	4	5
Actual						
0 WALKING	481	1	12	2	0	0
1 WALKING_UPSTAIRS	8	448	11	4	0	0
2 WALKING_DOWNSTAIRS	2	0	418	0	0	0
3 SITTING	3	6	0	442	40	0
4 STANDING	1	5	0	49	477	0
5 LAYING	0	0	0	0	0	537

**Table 5:** Performance of the CTM (*C* = 100, *T* = 9, *s* = 3.75) using a 9 × 114 patch size and a single Booleanization threshold on different modalities.

Rank	Combination Name	Channels	No. of Channels	Train Accuracy, %	Test Accuracy, %	Precision Weighted, %	Recall Weighted, %	F1 Weighted, %
1	*body_acc_xyz_* *total_acc_xyz_* *body_gyro_xyz*	*body_acc_x*, *body_acc_y*, *body_acc_z*, *total_acc_x*, *total_acc_y*, *total_acc_z*, *body_gyro_x*, *body_gyro_y*, *body_gyro_z*	9	95.42	91.45	91.65	91.45	91.30
2	*total_acc_xyz_* *body_gyro_xyz*	*total_acc_x*, *total_acc_y*, *total_acc_z*, *body_gyro_x*, *body_gyro_y*, *body_gyro_z*	6	93.74	88.66	89.11	88.66	88.43
3	*body_acc_xyz_* *total_acc_xyz*	*body_acc_x*, *body_acc_y*, *body_acc_z*, *total_acc_x*, *total_acc_y*, *total_acc_z*	6	93.36	87.58	87.98	87.58	87.32
4	*body_acc_xyz_* *body_gyro_xyz*	*body_acc_x*, *body_acc_y*, *body_acc_z*, *body_gyro_x*, *body_gyro_y*, *body_gyro_z*	6	93.64	84.66	85.28	84.66	84.89
5	*total_acc_xz*	*total_acc_x*, *total_acc_z*	2	86.61	79.74	80.18	79.74	79.33
6	*total_acc_xyz*	*total_acc_x*, *total_acc_y*, *total_acc_z*	3	88.00	78.41	78.40	78.41	77.90
7	*total_acc_xy*	*total_acc_x*, *total_acc_y*	2	86.58	78.18	79.18	78.18	77.85
8	*body_acc_xyz*	*body_acc_x*, *body_acc_y*, *body_acc_z*	3	86.61	77.50	78.06	77.50	77.66
9	*total_acc_x*	*total_acc_x*	1	76.68	73.70	81.40	73.70	67.68
10	*body_acc_xy*	*body_acc_x*, *body_acc_y*	2	81.86	72.51	73.93	72.51	73.02
11	*body_acc_xz*	*body_acc_x*, *body_acc_z*	2	78.46	71.76	72.12	71.76	71.80
12	*body_gyro_xyz*	*body_gyro_x*, *body_gyro_y*, *body_gyro_z*	3	82.90	71.05	71.16	71.05	71.00
13	*body_gyro_xz*	*body_gyro_x*, *body_gyro_z*	2	78.06	64.23	64.69	64.23	64.32
14	*body_gyro_xy*	*body_gyro_x*, *body_gyro_y*	2	76.38	62.60	62.75	62.60	62.50
15	*body_acc_x*	*body_acc_x*	1	66.25	61.82	61.98	61.82	60.65
16	*body_acc_yz*	*body_acc_y*, *body_acc_z*	2	73.21	60.29	60.19	60.29	58.93
17	*body_gyro_yz*	*body_gyro_y*, *body_gyro_z*	2	71.34	58.56	58.30	58.56	58.15
18	*total_acc_yz*	*total_acc_y*, *total_acc_z*	2	61.77	55.00	52.66	55.00	51.17
19	*body_acc_y*	*body_acc_y*	1	60.13	48.72	48.24	48.72	47.78
20	*body_gyro_z*	*body_gyro_z*	1	59.75	47.43	47.40	47.43	43.86
21	*total_acc_y*	*total_acc_y*	1	54.21	47.40	42.41	47.40	39.77
22	*body_gyro_y*	*body_gyro_y*	1	58.062	47.20	46.38	47.20	45.59
23	*body_acc_z*	*body_acc_z*	1	51.63	46.11	47.38	46.11	43.64
24	*body_gyro_x*	*body_gyro_x*	1	55.49	45.80	45.08	45.80	44.98
25	*total_acc_z*	*total_acc_z*	1	48.10	44.21	41.23	44.21	38.60

**Table 6:** Activity-wise F1 scores across inertial sensor modalities.

Rank	Combination Name	No. Channels	Activity-Wise F1 Score, %					
			WALKING	WALKING UPSTAIRS	WALKING DOWNSTAIRS	SITTING	STANDING	LAYING
1	*body_acc_xyz_* *total_acc_xyz_body_gyro_xyz*	9	93.50	96.99	94.83	79.20	85.31	98.53
2	*total_acc_xyz_body_gyro_xyz*	6	89.83	92.39	89.95	75.41	84.34	98.44
3	*body_acc_xyz_total_acc_xyz*	6	90.28	89.30	94.55	70.72	81.19	98.44
4	*body_acc_xyz_body_gyro_xyz*	6	93.80	94.47	98.34	68.67	76.75	80.63
5	*total_acc_xz*	2	84.75	82.68	84.25	56.04	69.56	98.53
6	*total_acc_xyz*	3	67.32	80.75	75.67	61.76	81.13	98.44
7	*total_acc_xy*	2	69.95	77.07	71.65	65.24	81.55	98.53
8	*body_acc_xyz*	3	89.31	86.15	96.34	55.89	65.19	77.12
9	*total_acc_x*	1	85.71	73.07	77.70	0.41	69.14	98.53
10	*body_acc_xy*	2	82.08	80.93	94.75	52.40	63.11	69.40
11	*body_acc_xz*	2	88.06	82.73	94.93	43.81	55.77	70.56
12	*body_gyro_xyz*	3	89.18	85.84	80.46	56.11	54.34	63.92
13	*body_gyro_xz*	2	86.10	72.18	67.36	54.32	52.86	55.45
14	*body_gyro_xy*	2	86.18	69.72	64.08	51.98	52.27	52.78
15	*body_acc_x*	1	79.85	62.60	79.59	25.74	54.48	64.42
16	*body_acc_yz*	2	76.45	76.88	66.51	30.57	46.35	59.50
17	*body_gyro_yz*	2	77.20	79.37	65.21	32.62	42.98	54.77
18	*total_acc_yz*	2	17.78	54.42	58.44	33.33	64.65	76.45
19	*body_acc_y*	1	61.03	67.68	44.62	25.53	46.98	41.69
20	*body_gyro_z*	1	61.11	63.86	44.01	5.34	37.10	52.19
21	*total_acc_y*	1	13.77	31.40	51.18	6.12	59.52	73.39
22	*body_gyro_y*	1	68.93	62.69	40.13	26.02	24.29	52.29
23	*body_acc_z*	1	64.55	55.75	43.88	23.68	29.00	46.29
24	*body_gyro_x*	1	81.52	36.09	36.19	39.55	43.18	32.65
25	*total_acc_z*	1	41.81	34.42	40.21	0.00	43.11	68.88

## Data Availability

The UCI-HAR dataset used in this study is publicly available at https://archive.ics.uci.edu/dataset/240/human+activity+recognition+using+smartphones (accessed on 24 May 2026). The Python (v. 3.14) implementation of the basic *MultiClassConvolutionalTsetlinMachine2D* from the *pyTsetlinMachineParallel* package (v. 0.2.1) is available at https://github.com/cair/pyTsetlinMachineParallel (accessed on 24 May 2026).
